# Worsening of Oxidative Stress, DNA Damage, and Atherosclerotic Lesions in Aged LDLr^−/−^ Mice after Consumption of Guarana Soft Drinks

**DOI:** 10.1155/2019/9042526

**Published:** 2019-06-10

**Authors:** Layla Aparecida Chisté, Beatriz Peters Pereira, Marcella Leite Porto, Jairo Pinto de Oliveira, Arícia Leone Evangelista Monteiro de Assis, Breno Valentim Nogueira, Silvana Santos Meyrelles, Tadeu Uggere de Andrade, Manuel Campos-Toimil, Elisardo Corral Vasquez, Bianca Prandi Campagnaro, Thiago Melo Costa Pereira

**Affiliations:** ^1^Pharmaceutical Sciences Graduate Program, Vila Velha University (UVV), Vila Velha, ES, Brazil; ^2^Federal Institute of Education, Science, and Technology (IFES), Vila Velha, ES, Brazil; ^3^Laboratory of Cellular Ultrastructure Carlos Alberto Redins (LUCCAR), Department of Morphology, Health Sciences Center, Federal University of Espirito Santo (UFES), Vitoria, ES, Brazil; ^4^Laboratory of Translational Physiology, Health Sciences Center, Federal University of Espirito Santo, Vitoria, ES, Brazil; ^5^Pharmacology of Chronic Diseases (CDPHARMA), Molecular Medicine and Chronic Diseases Research Center (CIMUS), University of Santiago de Compostela, Santiago de Compostela, Spain

## Abstract

**Background:**

Excessive consumption of soft drinks (SD) has become a health problem worldwide due to its association with related cardiovascular diseases. We investigated the possible impacts associated with the consumption of Brazilian guarana (normal and zero) SD in dyslipidemic mice, thus mitigating potential clinical confounders such as poor-quality diet, lifestyle, body composition, and/or comorbidities.

**Methods:**

Sixteen-month-old LDLr^−/−^ mice were divided into the following groups: (1) control; (2) GSD: normal guarana SD; and (3) Z-GSD: zero guarana SD. All were fed *ad libitum*, and blood pressure was measured noninvasively. After 8 weeks, aorta, blood, liver, and stomach samples were collected for histological and biochemical analyses.

**Results:**

Guarana soft drinks increased atherosclerosis (~60%) and were associated with hypercholesterolemia, hypertension, oxidative stress, DNA fragmentation, and apoptosis (~2-fold) of blood cells, besides presenting an increase in liver and gastric damage even in normoglycemia. Interestingly, Z-GSD did not cause the aforementioned changes, except in hemodynamic and renal parameters.

**Conclusions:**

Chronic administration of GSD is prooxidative, compromising the cardiovascular, gastric, and hepatic systems; the effects are due at least in part to free sugar consumption but not to guarana extract per se.

## 1. Introduction

Excessive consumption of sugar-sweetened soft drinks (SD) has become an alarming public health problem worldwide due to its association with dyslipidemia, weight gain, diabetes type 2, and other related cardiovascular diseases [1, 2]. As an alternative, SD containing artificial sweeteners, considered as “healthier,” have emerged to try to maintain the profitability of companies [3, 4]. However, the intrinsic toxicity of these carbonated beverages is still controversial, reinforced by scarce experimental data and potential clinical confounders, such as poor-quality diet, lifestyle, body composition, and/or comorbidities [5–7]. Thus, experimental studies are needed to clarify the impact of long-term consumption of SD and its consequences.

Interestingly, only in the last decade have some experimental studies described pathophysiological metabolic alterations after chronic exposure to sugar and artificially sweetened SD, especially colas [2, 8–13]. However, these data may not be extrapolated to all SD, as specific substances are present only in cola beverages (e.g., natural flavorings, colorants, fluid extract of coca, and phosphoric acid), and it is necessary to investigate other SD consumed in the world, such as guarana SD. Guarana SD are made from extracts that have been obtained from the dried seeds of guarana (*Paullinia cupana* H.B.K., Sapindaceae) in the Brazilian Amazonian region since 1921 [14, 15]. In the global market, guarana SD, available in both sugar and artificially sweetened forms, are among the fifteen highest selling SD in the world and the second most consumed in Brazil [16]. Until now, the possible consequences of long-term guarana consumption on cardiovascular-metabolic diseases have not yet been evaluated. Thus, this study reports for the first time the effects of long-term consumption of regular guarana SD (sucrose-sweetened, GSD) and zero guarana SD (aspartame-acesulfame K-sweetened, Z-GSD) in the LDLr^−/−^ mouse model.

As rats are commonly resistant to the effects of overabundant nutrients, genetically modified mice have been widely used in studies of cardiovascular-metabolic diseases, thus expanding translational applications [17–21]. Among these, LDLr^−/−^ mice show age-dependent susceptibility to concomitant metabolic complications such as dyslipidemia, obesity, and insulin resistance, mimicking humans with metabolic syndrome [21–23].

In this context, the objective of this study was to investigate possible biochemical and/or morphofunctional impacts associated with the consumption of GSD and Z-GSD in aged dyslipidemic mice, mitigating the influence of potential confounders associated with SD consumption in human subjects. These new results may help to fill the data gaps to better inform scientists, patients, clinicians, and governments.

## 2. Methods

### 2.1. Animals and Experimental Design

Under a normal chow diet provided *ad libitum*, female LDLr^−/−^ mice, aged ~16 months old, were randomly separated into 3 groups with free access to one of the following drinks: water (control), regular guarana SD (GSD) (sucrose-sweetened carbonated drink, Guaraná Antarctica®, Brazil), or zero guarana SD (Z-GSD) (low-calorie aspartame–acesulfame K-sweetened carbonated drink, Guaraná Antarctica Zero®, Brazil). The CO_2_ content was completely removed from both guarana drinks by vigorous shaking using a magnetic bar and a stirring plate in a container filled with the SD, after which the SD was offered to the animals at room temperature. Body weight and food and drink consumption were measured weekly. Cumulative food intake was calculated from the difference in weight before and after feeding. The average caloric food consumption was determined proportionally to food intake under the classic nutritional parameters: carbohydrate (4 kcal/g), protein (4 kcal/g), and fat (9 kcal/g).

Animals were bred and maintained in the animal care facility at the Experimental Monitoring Laboratory of Vila Velha University (UVV) under standard conditions in individual acclimatized plastic cages at ~22°C and 60% humidity under a 12-hour dark-light cycle. After 8 weeks of the diets, all animals were euthanized. All experimental procedures were performed in accordance with the guidelines for the care and handling of laboratory animals as recommended by the National Institutes of Health (NIH), and all protocols were approved by the Institutional Animal Care Committee (Protocol # 375/2016).

### 2.2. Blood Pressure Measurements

Systolic, diastolic, and mean blood pressure (BP) were measured using plethysmography of the tail (CODA Mouse Tail-Cuff Blood Pressure System, Kent Scientific Co., Connecticut, USA). Conscious mice were placed individually in a restraint that allowed free access to the tail and acclimated at 30°C for approximately 15 min to ensure adequate blood flow to the tail. BP measurements were performed in the morning in a quiet laboratory, and the mice were kept calm and handled by the same observer. Thirty-five measurements were recorded over 15 min, and valid readings (at least 10) were averaged to determine the values for BP. All measurements were recorded after a 3-day measurement acclimatization schedule on the first and last day of the diet.

### 2.3. Oral Glucose Tolerance Test (OGTT)

On the last day of the diet, basal glycemia was measured after 6 hours of total food and drink deprivation according to Andrikopoulos et al. [24] and Ayala et al. [25]. Fasted mice were orally administered 2 g of glucose/kg body weight, and blood glucose was checked through tail blood extraction at regular intervals (0, 20, 40, 60, and 120 min) as graphically indicated. For this, animals were placed in a restraint, and the last 4 mm of the tail was covered with clean gauze swabbed with lidocaine cream (4%). After 2 min, the anesthetic was removed with ethanol solution (70%), and the last 1 mm of the tip of the tail was then removed using sterilized surgical scissors. Then, the tail was gently massaged to ensure adequate blood flow. The total glucose response vs. time was evaluated by area under the curve (AUC) using Prism software (Prism 6.0, GraphPad Software Inc., San Diego, CA, USA).

### 2.4. Clinical Biochemistry Parameters

The blood was collected from the heart (right ventricle) of mice euthanized with sodium thiopental (100 mg/kg, i.p.). The blood was then centrifuged at 4,000 g for 10 min. Then, the serum was separated and kept at −80°C until analysis. Serum concentrations of glucose, triglycerides, total plasma cholesterol, high-density lipoprotein (HDL), uric acid, urea, creatinine, C-reactive protein (CRP), and homocysteine and the activity of AST and ALT were determined by an automatic biochemical analyzer (AU 680, Olympus/Beckman Coulter, Munich, Germany) according to the manufacturers' instructions. Standard controls were run before each determination. The levels of non-HDL lipoprotein were calculated by subtracting HDL from total serum cholesterol.

For the determination of enzymuria, as described by Fang et al. [26], urine samples were collected before euthanasia for the measurement of glutamyl transpeptidase (GGT) and creatinine. The samples were kept at −80°C before being assayed. All measurements were performed by standard laboratory methods using the same automatic biochemical analyzer.

### 2.5. Morphological Analysis of Aortic Lipid Deposition

The analysis of aortic lipid deposition was performed as previously described [20, 21, 27]. After euthanasia and venous blood collection, the animals were perfused with PBS-formaldehyde (4%; pH 7.4; 0.1 mol/L) (Merck S.A., São Paulo, Brazil) via the left ventricle, and the thoracic cavity was opened. Briefly, *en face* aortic surfaces were opened, fixed in ethylene vinyl-acetate (EVA), and stained with Oil Red O (Sigma-Aldrich, St. Louis, MO) to identify neutral lipids; images were captured using a digital camera. Then, quantification was performed using imaging software (ImageJ 1.35d, USA, public domain software from National Institutes of Health). Finally, the aortic lesion area was measured by a “blind” investigator.

### 2.6. Measurement of Oxidative Stress in the Blood (ROS Production)

ROS were quantified by flow cytometry analysis according to previous protocols [21, 28]. To estimate the bioavailability of intracellular superoxide (^•^O_2_^−^) and hydrogen peroxide (H_2_O_2_), dihydroethidium (DHE, 160 *μ*M) and 2′,7′-dichlorofluorescein diacetate (DCF, 20 mM) were, respectively, added to a cell suspension (10^6^ cells) and incubated for 30 min (at 37°C) in the dark. In relation to highly reactive oxygen species (hROS), such as peroxynitrite and hydroxyl radicals, they were selectively measured using HPF (2-[6-(4′-hydroxy)phenoxy-3H-xanthen-3-on-9-yl] benzoic acid). After washing the cells and resuspension in PBS, the samples were analyzed using a flow cytometer (FACSCanto II, BD Biosciences, San Jose, CA). All the data were obtained through the FACSDiva software (BD Biosciences, San Jose, CA), and histograms were generated using the FCS Express software (De Novo Software, Thornhill, Ontario). For the measurement of the probes DHE, DCF, and HPF fluorescence, ten thousand events per sample were explored for each analysis in monoplicate with excitation occurring at 488 nm. For ^•^O_2_^−^ quantification, DHE fluorescence was measured through a bandpass filter at an excitation/emission wavelength of 585/42 nm, whereas DCF and HPF fluorescence were analyzed through 530/30 nm. Finally, the results were reported as the median fluorescence intensity (MFI).

### 2.7. Cell Viability

This protocol was analyzed through propidium iodide (PI) as previously described [29]. A total of one million cells were exposed to PI (2 *μ*L) in the medium (5 min at 25°C in the dark).

After the washing step with PBS, the blood cells were submitted to flow cytometry using a FACSCanto II Flow Cytometer (BD Biosciences). For viability analysis, samples were obtained in triplicate. A total of 10,000 events were analyzed for each experiment at 488 nm excitation whereas PI fluorescence was observed through a bandpass filter at 585/42 nm. All the data are represented as the proportion (%) of unstained/viable cells [29].

Apoptotic blood cells were analyzed according to the protocol of our lab (Porto et al. [28] and Bôa et al. [29]). In brief, cells were washed two times with PBS and adjusted to 0.5 mL with the binding buffer (500,000 cells). After the incubation with annexin V-FITC and PI (at 25°C in the dark for 15 min), the cells were detected using a FACSCanto II (BD Biosciences) flow cytometer. Apoptotic cells were identified by the positive staining for annexin V (*Q*2 + *Q*4).

### 2.8. Advanced Oxidation Protein Products (AOPP) in Plasma, Liver, and Stomach

The analyses of AOPP were performed according to Witko-Sarsat et al. [30] and Coutinho et al. [21] using spectrophotometry with a microplate reader (SpectraMax 190, Molecular Devices, Sunnyvale, CA, USA). Forty microliters of plasma, liver, or stomach homogenate (diluted at 1:  10, 1:  30, and 1:  10, respectively) was solubilized 1:  5 in PBS or chloramine-T standard solutions (0 to 100 *μ*mol/L). Then, the samples were placed in each well of a 96-well microtiter plate (BD Discover Labware, Lincoln Park, NJ, USA), and 10 *μ*L of 1.16 mol/L potassium iodide (KI, Sigma-Aldrich) was added, followed by the addition of 20 *μ*L of acetic acid. The absorbance of the reaction mixture was immediately read at 340 nm in a microplate reader against a blank containing 200 *μ*L of PBS, 10 *μ*L of KI, and 20 *μ*L of acetic acid. Finally, the AOPP was determined when the correlation coefficient was >0.95. The concentrations were presented in *μ*mol/mg of total protein as determined by the Bradford method [31] from dilutions of 1:  200 for plasma, 1:  50 for liver, and 1:  4 for stomach for each measurement.

### 2.9. Comet Assay

DNA damage was assessed using an alkaline comet assay (single-cell gel electrophoresis), based on Singh et al. [32] following minor adaptations established from our laboratory [33, 34]. Briefly, histological slides were precoated with 1.5% normal melting point agarose in water in a water bath at 65°C. Subsequently, 10 *μ*L of blood cell suspension was embedded in 110 *μ*L of 1% low melting point agarose in PBS at 37°C and spread on agarose-precoated slides using coverslips. The experiment was conducted in duplicate, i.e., two slides per animal. After gelling at 4°C for 30 min, the coverslips were removed, and the slides were incubated in freshly prepared lysis solution (in mmol/L: 2500 NaCl, 100 EDTA, 10 Tris, and 34 N-lauroylsarcosine sodium, adjusted to pH 10.0-10.5, using freshly added 1% Triton X-100 and 10% DMSO) for 1 hour at 4°C. Then, the slides were placed in an electrophoresis chamber filled with freshly prepared alkaline buffer (in mmol/L: 300 NaOH and 1 EDTA, pH >13) for 40 min at 4°C and conducted at 300 mA and 32 V (1 V/cm) for 20 min. Afterwards, the slides were neutralized with a 0.4 mol/L Tris buffer (pH 7.5) for 5 min (3 times) and finally dried with cold pure methanol (-20°C) for fixation. Migration of DNA fragments towards the anode creates a comet “tail,” visualized by staining with ethidium bromide (20 *μ*g/mL, Sigma-Aldrich). Immediately afterwards, images were obtained at a magnification of 20x using a fluorescence optical microscope (Eclipse TI, Nikon Instruments Inc., Melville, NY, USA) equipped with excitation (420-490 nm) and barrier (520 nm) filters. The coded images were acquired using a CCD camera (Nikon) and were analyzed with the CASP 98beta program (public domain).

Among the several parameters provided by the CASP program, we used the percentage of DNA in the tail and the tail moment for analysis of DNA damage. The images of 50 randomly selected nucleoids from each sample were analyzed for each animal. During the image analysis, nucleoids without clearly identifiable heads, showing overlap, or containing an artifact were excluded as a quality control measure.

### 2.10. Liver and Stomach Histology

For detection of neutral lipids for morphometric analyses, the organs were isolated and fixed in buffered formaldehyde solution (4%) for at least 2 days. The samples were cross-sectioned at 8 *μ*m thicknesses in a -25°C cryostat (Jung CM1860; Leica, Wetzlar, Germany). Sections were then mounted on gelatin-coated slides and colored with Oil Red O or hematoxylin-eosin (Sigma-Aldrich). Finally, all images were captured using a camera (AxioCam ERc 5 s, Carl Zeiss, Germany) coupled to an optical microscope (AX70, Olympus Corporation, Japan) with a 40x objective and quantified using ImageJ software (NIH, USA). For each analysis, 10 distinct fields per animal were randomly used to calculate the average percentage of the red area. All analyses were performed by a “blind” researcher.

### 2.11. Statistical Analysis

All data are expressed as the mean ± SEM (standard error of the mean). For the statistical analysis, two-way ANOVA was performed to analyze differences in biometric parameters and chow, liquid, and caloric intake. For the other analyses, we used one-way ANOVA followed by post hoc Tukey's test using Prism software (Prism 6.0, GraphPad Software Inc., San Diego, CA, USA). A value of *p* < 0.05 was considered statistically significant.

## 3. Results

### 3.1. Biometric Parameters and Chow, Liquid, and Caloric Intake

**Figure 1:**
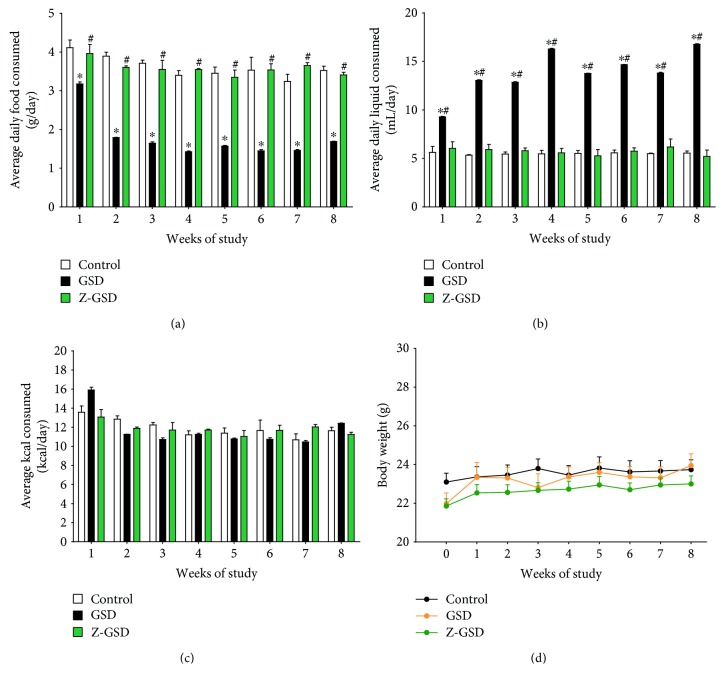
Variation in nutrient intake and body weight of LDLr^−/−^ mice after treatment with guarana soft drinks (normal and zero) for 8 consecutive weeks. (a) GSD mice consumed less food on average daily than control or Z-GSD-fed mice during 8 consecutive weeks (^∗^*p* < 0.05). (b) On the other hand, the GSD group presented polydipsia compared to the control (^∗^*p* < 0.05) and Z-GSD groups (^#^*p* < 0.05). (c) There were no differences in caloric intake between groups. (d) During this period, transient changes in body weight in response to GSD or Z-GSD intake were not observed (two-way ANOVA with repeated measures).

Figure 1 summarizes the general parameters of food and liquid intake, caloric consumption, and body weight during the 8-week follow-up period. The GSD group exhibited hypophagia (~50%, *p* < 0.05, Figure 1(a)) and polydipsia (~2.5-fold, *p* < 0.05, Figure 1(b)) when compared to the control and Z-GSD groups, without a difference in caloric consumption and body weight between groups.

### 3.2. Lipid Profile and En Face Analysis

**Figure 2:**
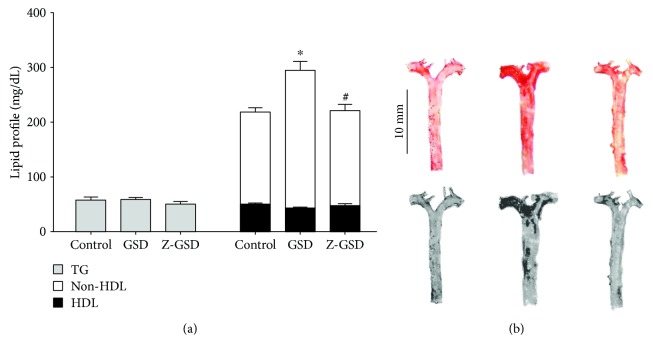
Plasma lipoprotein profiles in the three experimental groups of LDLr^−/−^ mice showing that guarana soft drinks (GSD) increase non-HDL cholesterol (~30%) and vascular lipid deposition (~60%) compared with the control and zero guarana soft drink (Z-GSD) diets. (a) Bar graphs show the lipid profile between groups. (b) Representative aorta *en face* images of Oil Red O staining and their respective densitometry analyses. Bar graph depicting average vascular lipid deposition areas. Values are presented as the mean ± SEM for *n* = 6‐9 animals per group. ^∗^*p* < 0.05 vs. the control group and ^#^*p* < 0.05 vs. the GSD group (one-way ANOVA).

Figure 2 represents the serum lipid profile and lipid deposition in the aortas of all groups studied. We observed an augmentation in non-HDL cholesterol in GSD mice (252 ± 16 mg/dL, *n* = 10, *p* < 0.05) compared with the control and Z-GSD groups (168 ± 8, *n* = 9, and 174 ± 11 mg/dL, *n* = 10, respectively) without a difference in the level of HDL and triglycerides (Figure 2(a)).  Figure 2(b) summarizes the results of typical analyses of the aorta, showing that GSD mice had significantly increased lipid deposition, by ~60% (*p* < 0.05), compared with the control group (3.5 ± 0.5%). On the other hand, in the aortas obtained from Z-GSD mice, the lipid deposition was similar to that under control conditions (4.4 ± 0.4%, *p* > 0.05).

### 3.3. Other Serum Biochemical Parameters

Table 1 shows the results of other relevant biochemical analyses of the 3 groups studied after 8 weeks of the diets. Interestingly, we highlighted that the nonfasting glycemic level was not different between the groups. Surprisingly, in the OGTT, the GSD and Z-GSD groups showed augmented sensitivity to insulin (~30%) compared with control mice (*p* < 0.05). In parallel, the renal biomarkers worsened due to Z-GSD supplementation: serum creatinine increased significantly (~65%, *p* < 0.05) in the Z-GSD group compared to the control and GSD groups. Moreover, the GGT/urinary creatinine was also higher in the Z-GSD group than in the control and GSD groups (~100 and 65%, respectively, *p* < 0.05). No differences were observed between hepatic (ALT and AST) and nonspecific inflammatory (CRP) biomarkers.

### 3.4. Blood Pressure Determination

**Figure 3:**
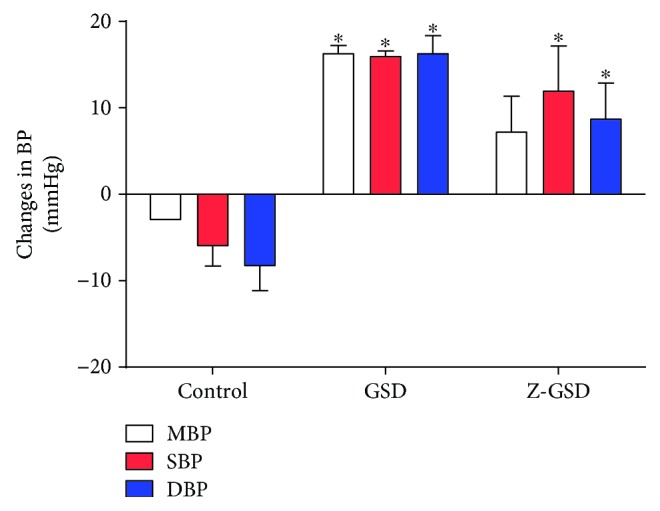
Variations in hemodynamic parameters of mean, systolic, and diastolic blood pressure (MBP, SBP, and DBP, respectively) among 3 experimental groups of LDLr^−/−^ mice. The graph demonstrates that both normal (GSD) and zero (Z-GSD) guarana soft drinks increase the absolute BP over 8 consecutive weeks. Values are presented as the mean ± SEM for *n* = 3‐5 animals per group. ^∗^*p* < 0.05 vs. the control group (one-way ANOVA).

As shown in Figure 3, the GSD group exhibited significant increases in mean, systolic, and diastolic BP (+19, 22, and 24 mmHg, respectively, *p* < 0.05) compared with the control group. On the other hand, the Z-GSD mice showed increases in only systolic and diastolic BP (18 and 17 mmHg, respectively, *p* < 0.05). No change in heart rate was observed between groups (data not shown).

### 3.5. Oxidative Stress Biomarkers in Blood Cells

**Figure 4:**
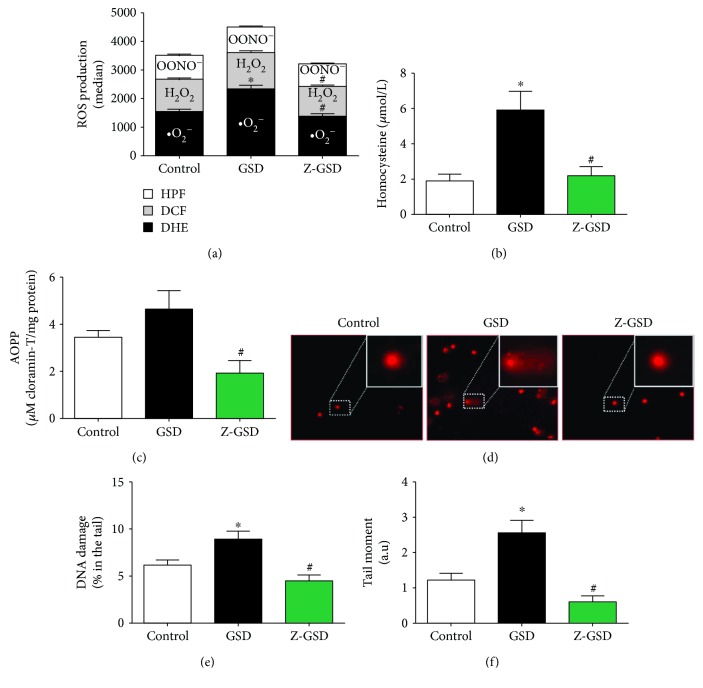
ROS overproduction and genotoxic effects are associated with normal guarana soft drink (GSD) but not in zero guarana soft drink (Z-GSD) intake. (a) ROS production was assessed by DHE, DCF, and HPF and measured by flow cytometry. (b) Quantification of homocysteine and (c) protein oxidation (AOPP) in the serum of the 3 groups. (d) Detection of DNA damage in blood is assessed by an alkaline comet assay. Typical comets show greater DNA fragmentation only in the GSD group compared to that in the control group, which contrasts with the Z-GSD group, quantified and represented in the graph in (e). Bar graph in (f) shows the percentage of DNA tail moments (~2-fold higher in the GSD group). Values are presented as the mean ± SEM for *n* = 6‐7 animals per group. ^∗^*p* < 0.05 vs. the control group and ^#^*p* < 0.05 vs. the GSD group (one-way ANOVA).

Based on previous data showing that high levels of ROS are crucial for atherosclerosis [19, 21, 35–37] and hypertension [38], we evaluated the intracellular ROS levels in white blood cells in all groups studied. As illustrated in Figure 4(a), we showed that GSD supplementation increased ^•^O_2_^−^ production by ~50% (2, 333 ± 135 a.u.) compared to that in the control mice (^•^O_2_^−^: 1, 544 ± 85 a.u., *p* < 0.05). Interestingly, Z-GSD prevented the overproduction of ROS (^•^O_2_^−^: 1, 388 ± 79, H_2_O_2_: 1, 038 ± 48), producing levels similar to that in the control group (H_2_O_2_: 1, 140 ± 41, *p* > 0.05). In relation to hROS, we did not detect differences between groups (control: 831 ± 41, GSD: 897 ± 30, Z-GSD: 792 ± 29 a.u., *p* > 0.05). Concerning other serum biomarkers of oxidative stress, we demonstrated that the GSD group had increased plasma homocysteine (~3-fold, Figure 4(b)) and AOPP levels (~2.5-fold, Figure 4(c)) compared to the Z-GSD group (*p* < 0.05).

### 3.6. Genotoxic Effect

The assessment of genotoxic stress by the comet assay indicated greater DNA damage in the GSD group (8.9 ± 0.9%, *p* < 0.05) than in control mice (6.2 ± 0.5%), and this damage was significantly reduced in the Z-GSD group (4.5 ± 0.6%) (Figures 4(d) and 4(e)). Another parameter measured was the comet tail moment, the product of the tail length and the portion of total DNA in the tail [34, 39]. This analysis demonstrated an increase in DNA fragmentation in the GSD group (2.6 ± 0.35 a.u., *p* < 0.05) compared with the control and Z-GSD mice (1.2 ± 0.2 and 0.6 ± 0.2 a.u., respectively) (Figure 4(f)).

### 3.7. Cell Viability and Apoptosis in Blood Cells

**Figure 5:**
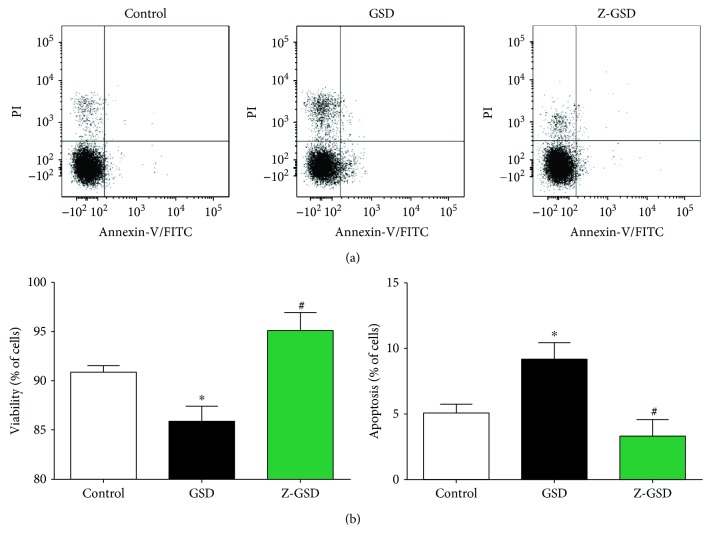
Normal (GSD) but not zero (Z-GSD) guarana soft drink intake increases apoptosis and compromises cell viability in blood cells. (a) Dot plots showing the apoptosis ratios from the control, GSD, and Z-GSD groups (*n* = 5). (b) The apoptosis and cell viability ratios were determined using propidium iodide (PI) and FITC-annexin V. The *Q*2 + *Q*4 quadrants represent the cells that are in apoptosis. Note the remarkable decrease in the number of apoptotic cells (*Q*2 + *Q*4) in the Z-GSD group. Values are presented as the mean ± SEM. ^∗^*p* < 0.05 vs. the control group and ^#^*p* < 0.05 vs. the GSD group (one-way ANOVA).

Apoptosis was investigated in the same blood cells using PI and annexin V staining and flow cytometry analysis.  Figure 5(a) shows typical dot plots for each group. Our results in Figure 5(b) indicate that GSD increased the number of apoptotic cells (*Q*2 + *Q*4) by 80% (*p* < 0.05) compared with that of control mice (5.1 ± 0.7). On the other hand, the Z-GSD group showed a profile similar to that of the control mice (3.3 ± 1.2%, *p* < 0.05). Concerning cell viability (Figure 5(c)), the GSD group showed impaired cell viability (86 ± 1.6%) compared with that of the other groups (control: 91 ± 0.7% and Z-GSD: 95 ± 1.8%, *p* < 0.05).

### 3.8. Oxidized Protein and Histological Analysis in Liver and Stomach

**Figure 6:**
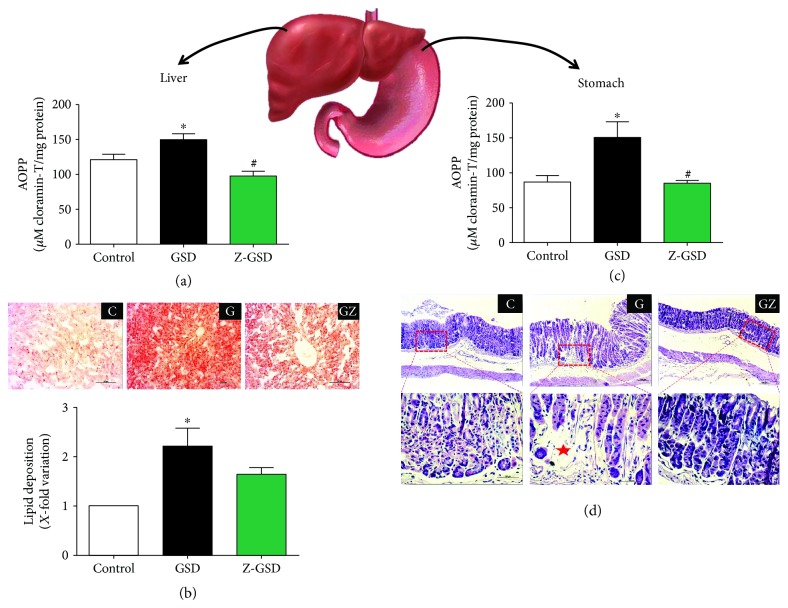
Chronic normal (GSD) but not zero (Z-GSD) guarana soft drink intake increases liver and stomach damage. (a) GSD intake augments advanced oxidation protein products (AOPP) and (b) lipid deposition in liver, illustrated through representative images in the top panel. (c) Similarly, GSD increases AOPP formation in the stomach. (d) Only the GSD group developed atrophy and degeneration in gastric glands. The values are presented as the mean ± SEM for *n* = 4 − 6 animals per group. ^∗^*p* < 0.05 vs. the control group and ^#^*p* < 0.05 vs. the GSD group.

We also investigated the impact of chronic guarana consumption on oxidative damage in the liver and stomach of LDLr^−/−^ mice. The levels of oxidized proteins were increased in the GSD group compared to control animals, in both the liver (25%, *p* < 0.05, Figure 6(a)) and stomach (75%, *p* < 0.05, Figure 6(c)). Interestingly, the Z-GSD group and the control mice had similar profiles (*p* > 0.05) in both organs (Figures 6(a) and 6(c)). Moreover, the GSD group showed greater lipid deposition (120%, *p* < 0.05) in liver cells (Figure 6(b)) compared with that of the control group. The Z-GSD group showed no difference compared to the other groups (*p* > 0.05). In regard to stomach damage (Figure 6(d)), we observed that only the GSD group developed atrophy and degeneration in gastric glands.

## 4. Discussion

In the present study, we showed for the first time that long-term consumption of the regular classic guarana beverage (GSD) by adult dyslipidemic mice resulted in an increase in hypercholesterolemia, aortic lipid deposition, BP, oxidative stress, and DNA fragmentation, as well as apoptosis in mononuclear cells, and greater hepatic and gastric injuries (even without weight gain or hyperglycemia). Interestingly, the zero guarana soft drinks (Z-GSD) did not cause most of the described negative effects, except those on hemodynamic and renal parameters.

First, although several clinical studies have indicated a positive association between sugar-sweetened beverage consumption and the risk of obesity [40–42], our results, as well as other related experimental findings [1, 12, 43], have not confirmed the clinical hypothesis that the regular consumption of sugar-sweetened beverages could induce weight gain. It is important to emphasize that, contrary to rational human behavior, the animals exposed to classic guarana halved their consumption of food, thereby normalizing their caloric intake, as previously described by Otero-Losada et al. [12], who investigated cola beverage consumption in the same exposure period. Though some researchers suggest that consumption of nonnutritive sweeteners may increase appetite [10, 44, 45], our results using zero guarana SD do not show modified food intake or body weight. Therefore, our data reinforce recent experimental findings using diet cola, which had similar results [10–12], and suggest that an increase in food consumption associated with aspartame-sweetened drinks in humans might be related to psychological influences (eating in excess) that apparently did not occur in our experimental animals.

It is well documented that consumption of SD is linked to cardiometabolic risk factors [46–48]. However, the parameters of the traditional serum biochemical profile (e.g., glycemia, triglycerides, and cholesterol) under SD exposure are still conflicting in experimental [1, 10, 12] and clinical studies [45–51]. Our results showed that GSD, but not Z-GSD, increased only non-HDL cholesterol, maintaining triglyceride and glycemic control. A possible explanation for the euglycemic control is that drinks containing a moderate amount of caffeine [1, 8, 52] and/or acesulfame K [12, 53] might stimulate insulin secretion and/or upregulate glucose transporters, which is partially corroborated by the improvement of the OGTT profile detected in our study. Despite the discrete metabolic impact observed, we demonstrated several consequences of long-term nonzero guarana consumption, described as follows.

The exact influence of chronic SD consumption on atherosclerosis is poorly known. Until now, experimental data have been collected only for cola beverages [12, 54]. Therefore, the proatherogenic effect observed with GSD (but not with Z-GSD) opens new perspectives about this issue, justified by the following points: (1) it is possible to induce significant aortic lipid deposition in aged female LDLr^−/−^ mice under only sugar-sweetened beverage exposure without a high-fat diet, which opposes the classical methodology [21, 55]; (2) the atherogenesis might be more related to the excessive exposure to free sugars than other substances (nonnutritive sweeteners) in these SD, as observed in a study using cola beverages that detected atherogenesis after exposure to even light cola drinks [54]; and (3) glycemia per se may not be sufficient to evaluate the impact of chronic exposure to carbohydrate-rich beverages, verified by hemodynamic parameters, redox homeostasis, and cellular/tissue losses, as detected in our study.

Concerning hemodynamic parameters, several reports have shown that hypertension is a major contributor to the development of cardiovascular diseases, which are associated with endothelial dysfunction and altered contractility [27, 56, 57]. For the first time, our study demonstrated an increase in BP due to chronic guarana SD consumption, which could be involved in the development of hypertension. Although we and others have not yet explored the vascular reactivity of animals exposed to classic SD, some studies have previously shown abnormal reactivity in animals fed a high fructose diet [58, 59], justified at least in part by an increase in angiotensin II and downregulation of eNOS [60]. On account of the present data, we suggest that other substances may be involved in the potential development of hypertension because the group treated with an artificially sweetened drink (Z-GSD) also showed a rise in BP. Among them, we cannot discard the role of aspartame (which contains 50% phenylalanine), a relevant precursor of highly vasoactive substances (i.e., dopamine, noradrenaline, and adrenaline) [61], and caffeine, an enhancer of adrenergic activity [10].

It is well established that oxidative stress is a central phenomenon in the progression of cardiovascular [19, 21, 23, 27, 35, 62] and other age-related diseases [63, 64]. At the same time, several studies have shown that hyperglycemia increases ROS production mainly via mitochondrial dysfunction and endoplasmic reticulum stress [28, 65–67]. Therefore, we decided to investigate the impact of ROS generation and possible cell/tissue oxidative damage under exposure to guarana drinks. For the first time, we showed that classic guarana soft drinks (GSD) present a prooxidative effect by both direct (flow cytometry) and indirect (homocysteine, AOPP, and DNA fragmentation) detection and that all these factors may influence, at least in part, the increase in BP and the lipid deposition observed in our study [35, 68–70]. Additionally, we previously showed that oxidative damage had major consequences, such as elevation of apoptosis and decrease of blood cell viability, whose mechanisms may be through established pathways (e.g., chromosomal cleavage, telomere shortening, and/or activation of caspases) [71–73]. Moreover, we cannot exclude the possibility that hyperhomocysteinemia observed under exposure to guarana might be another direct contributor to hypertension [64], DNA damage, and apoptosis activation, as observed by others [74, 75].

Until the present study, there was no evidence that guarana SD could contribute to liver or gastric damage. In parallel, only a few studies have demonstrated a potential risk of hepatotoxicity under chronic exposure to aspartame [76, 77] or acesulfame K [12]. Thus, our data clarify this question by demonstrating that the excessive consumption of carbohydrates (but not guarana per se) is the main contributor to steatosis and oxidative stress in the liver. These data are supported by previous research showing that fructose and glucose are important inducers of de novo lipogenesis and ROS production [78, 79]. A similar pattern of impacts was observed for the stomach, revealing that the greatest damage occurs in relation to carbohydrates (fructose, glucose, and sucrose) rather than to artificial sweeteners (acesulfame K or aspartame). It is important to emphasize that all damages previously described were generated in normoglycemic conditions, even in the case of chronic exposure to classic SD (rich in carbohydrates). Thus, it is suggested that before classical metabolic alterations are observed clinically, several types of oxidative damage by SD may occur. This should be considered an alert relevant to public health policies.

Last, but not least, the only organ that showed greater damage from zero guarana SD was the kidney, with possible loss of glomerular filtration and tubular injury, demonstrated by creatininemia and increased enzymuria, respectively, according to previous reports [26, 80]. In this case, we suggest that the renal damage might have been generated by aspartame. Recently, some studies have demonstrated that long-term intake of aspartame may develop oxidative stress in the rat kidney through the dysregulation of glutathione homeostasis [81]. However, future investigations will be necessary to explore the impact of acesulfame K or other substances.

Some limitations of our investigation should be considered. First, the lack of monitoring the estrous cycle in adult female mice does not allow us to speculate about the influence of sex hormones on the present results. Second, since this study with SD was carried out for the first time with LDLr^−/−^ mice, the comparison with the ApoE^−/−^ mouse model would not be appropriate. Third, we do not yet know if all parameters would be normalized after washout for months, indicating the need for more investigations to confirm or rule out the existence of nonreversible, chronic effects after prolonged consumption of these beverages.

## 5. Conclusions

Our results demonstrate that long-term administration of the classic guarana beverage causes adverse prooxidant outcomes at serum, vascular, hepatic, and gastric levels, at least in part due to free sugar exposure but not to guarana extract per se. This experimental investigation may provide a basis for further experimental and clinical studies to better explore the association between the consumption of soft drinks and metabolic diseases.

## Figures and Tables

**Table 1:** Serum biochemical parameters in experimental groups of LDLr^−/−^ mice.

Parameters	Groups			*p*
	Control	GSD	Z-GSD	
Glucose (mg/dL)	206 ± 12	211 ± 19	196 ± 20	0.2856
OGTT (AUC_0-120_)	28476 ± 2159	20170 ± 1962^∗^	20830 ± 1552^∗^	0.0154
Uric acid (mg/dL)	2.8 ± 0.2	2.9 ± 0.2	3.0 ± 0.7	0.9577
Blood urea (mg/dL)	57 ± 4	57 ± 4	58 ± 4	0.9651
Serum creatinine (mg/dL)	0.20 ± 0.01	0.21 ± 0.02	0.33 ± 0.05^∗^^#^	0.0082
GGT/urinary creatinine (100 U/g)	1.66 ± 0.34	2.00 ± 0.46	3.34 ± 0.46^∗^	0.0323
ALT (U/L)	37 ± 3	41 ± 6	39 ± 8	0.8829
AST (U/L)	168 ± 29	250 ± 52	371 ± 134	0.2626
AST/ALT ratio	3.9 ± 0.5	4.4 ± 0.5	4.5 ± 0.6	0.7576
CRP (mg/L)	0.29 ± 0.03	0.39 ± 0.09	0.31 ± 0.04	0.4656

Note: the values are presented as the mean ± SEM for *n* = 7 to 13 animals per group. ^∗^*p* < 0.05 vs. the control group and ^#^*p* < 0.05 vs. GSD (guarana soft drink group). Z-GSD: zero guarana soft drink.

## Data Availability

All data used to support the findings of this study are included within the article.

## Abbreviations

ALT: Alanine aminotransferase
ANOVA: Analysis of variance
AOPP: Advanced oxidation protein products
AST: Aspartate aminotransferase
AUC: Area under the curve
BP: Blood pressure
FITC: Fluorescein isothiocyanate
GGT: Glutamyl transpeptidase
GSD: Normal/classic guarana soft drink
Z-GSD: Zero guarana soft drink
HDL: High-density lipoprotein
i.p.: Intraperitoneal
kcal: Kilocalories
LDLr^−/−^: Low-density lipoprotein receptor knockout mice
MFI: Median fluorescence intensity
min: Minutes
NIH: National Institutes of Health
OGTT: Oral glucose tolerance test
PBS: Phosphate-buffered saline
CRP: C-reactive protein
PI: Propidium iodide
ROS: Reactive oxygen species
SD: Soft drinks
SEM: Standard error of the mean
UVV: Vila Velha University.
